# Need for cognition, academic self-efficacy and parental education predict the intention to go to college—evidence from a multigroup study

**DOI:** 10.3389/fpsyg.2025.1487038

**Published:** 2025-02-05

**Authors:** Lina Kramer, Stefanie Lüdtke, Philipp Alexander Freund

**Affiliations:** ^1^Intensive Care Medicine, Amsterdam University Medical Centers, Amsterdam, Netherlands; ^2^Human Resources Department, Polizei Hamburg, Hamburg, Germany; ^3^Institute of Psychology in Education, Faculty of Education, Leuphana University Lüneburg, Lüneburg, Germany

**Keywords:** need for cognition, academic self-efficacy, academic ambition, college-going intentions, first-generation students

## Abstract

Academic success is not solely the result of cognitive ability. There is evidence that traits such as students' need for cognition (NFC) and self-efficacy beliefs influence academic success. Beyond cognitive ability and personal traits, social background constitutes an important factor. Students from academic households are (still) much more likely to pursue an academic degree than their peers from non-academic households. Past research on traits and beliefs relevant in (higher) education has focused on academic success, but only to a limited extent on its direct precursor: the decision to pursue an academic degree. This study aims to investigate NFC and academic self-efficacy (ASE) as positive predictors of students' intentions to go to college, with consideration of students' generational status regarding academic education. Results based on survey data from 1,389 German high school students provide evidence for positive relationships between NFC, ASE, and study intention, with ASE acting as a mediator of NFC's effect. Our analyses also investigate the effects of NFC and ASE on study intentions for students from academic as compared to students from non-academic households via multigroup analyses.

## Introduction

Academic success can be predicted reasonably well by cognitive abilities and broad personality traits, for instance, openness to experience (Deary et al., 2007; Trapmann et al., 2007). In higher education, the necessary precursor of academic success is the decision to enroll in programs offering higher academic education. By attempting to understand the potential interplay between selected narrow personality constructs related to educational prowess and the familial background of students as a social factor, we aim to broaden the scope of research on a matter relevant to the continued development of democratic societies, namely, individuals' intentions to obtain (degrees of formal) education, as expressed by academic success.

A useful approach to understanding interindividual differences in study intentions is to consider the rather general construct of (academic) self-concept. Integral parts of an individual's self-concept are their preferences and beliefs. Individuals vary in their preference for learning and the enjoyment they derive from it. These differences in engagement in and enjoyment of cognitive challenges are captured by intellectual investment traits such as need for cognition (Cacioppo and Petty, 1982; von Stumm, 2018). Moreover, individuals differ in their beliefs about their ability to operate successfully in academic settings. These beliefs are usually described by academic self-efficacy (Schunk, 1991). Both intellectual investment traits and academic self-efficacy may explain interindividual differences in study intentions.

Social background sets the context in which personal preferences and beliefs develop. It has repeatedly been shown that children of parents with academic degrees are much more likely to pursue higher education themselves than children of parents without higher academic education (OECD, 2018; for an empirical study, see, for instance, Jung and Lee, 2019). Differences in study intentions between students from academic and non-academic households may in part be due to differences in (acquired) personal beliefs and preferences.

We aim to investigate systematic differences in the degree of study intentions between students from academic and non-academic households as well as potential influences of intellectual investment traits and academic self-efficacy beliefs.

### Parental education

The “socioeconomic achievement gap” refers to the differences in academic success between students from backgrounds with high and low socioeconomic status (SES), which is commonly indicated by parental education and income (see e.g., White, 1982). This educational gap continues to persist in international assessments of student performance such as, for instance, the PISA studies (OECD, 2018). Indeed, for a majority of the PISA participating countries, the socioeconomic achievement gap has *increased* between 1964 and 2015 (Chmielewski, 2019). It has been suggested that this increase is due to the growing accessibility of academic education on all levels of the educational system, which means more people from different backgrounds are going further in the educational system and existing gaps have a better chance of being recorded. Inequity in educational attainment is in part a result of external factors such as the availability of study materials and social resources. For example, first-generation students (no parent with an academic degree) report lower social support from their friends and family than their peers who are not first-generation students (Jenkins et al., 2013). At the same time, there are differences in students' attitudes toward and beliefs about academic education which are partly contingent on family background. With particular attention to parental education, research has identified notable differences between students with parents who have tertiary academic education and students who would be the first in their family to pursue it.

First-generation students report lower educational aspirations (McCarron and Inkelas, 2006), are less likely to pursue STEM majors in college than their peers, and have a lower math and science self-concept of ability in high school (Jiang et al., 2020). They are also less likely to actually achieve their educational aspirations (McCarron and Inkelas, 2006). Moreover, first-generation students exhibit lower academic self-efficacy (van Rooij et al., 2017) and higher negative outcome expectations (Ramos-Sánchez and Nichols, 2007), and they expect more barriers if they were to attend college (Gibbons and Borders, 2010). Significant differences in the academic self-efficacy of students from academic and non-academic households persist even when grades do not differ between these groups (Mohrenweiser and Pfeiffer, 2016).

### Need for cognition

Intellectual investment traits play a role in the way individuals invest effort and time into developing their intellect (von Stumm et al., 2011). A major established intellectual investment trait is need for cognition (NFC), which is defined as an “individual's tendency to engage in and enjoy effortful cognitive activity” (Cacioppo and Petty, 1982, p. 116). NFC as a trait thus implies that some people desire deeper understanding of information than others. Meta-analytical findings show that NFC is positively related to cognitive ability and knowledge (von Stumm and Ackerman, 2013), academic achievement (Liu and Nesbit, 2024), and even (multiple) aspects of wellbeing (Zerna et al., 2024).

Notably, for college students, NFC has been found to be positively related to cognitive ability (Cacioppo and Petty, 1982), cognitive effort (Cacioppo et al., 1983), GPA, life satisfaction (Coutinho and Woolery, 2004), self-esteem (Epstein et al., 1996), study satisfaction and college retention (Grass et al., 2017). NFC has also been investigated as a mediator of the positive effect of openness on cognitive ability (Furnham and Thorne, 2013). Studies have suggested positive relations between NFC and not only academic outcomes but also situations of daily life. For instance, individuals who are high in NFC tend to engage more with others' ideas and opinions, along with being more willing to immerse themselves into topics that are not usually a focus of their personal interest (Strobel et al., 2018).

Regarding the relationship between NFC and social background, there are inconsistent findings to date. While Padgett et al. (2010) found no evidence for a direct relationship between intellectual investment traits and parents' educational status, van Rooij et al. (2017) did report high school students from non-academic households to score significantly lower in NFC than their peers.

There is evidence for NFC's incremental validity in predicting goal-oriented behavior and intentional resource allocation (Fleischhauer et al., 2010). NFC fits into the seeking process of intellectual engagement because people higher in NFC are more likely to seek out intellectually challenging situations (Mussel, 2013). Higher levels in intellectual investment traits are associated with the pursuit of environments that provide learning possibilities (von Stumm et al., 2011). Such tendencies presumably translate to higher study intentions.

### Academic self-efficacy

Self-efficacy describes the beliefs a person has about their ability to appropriately manage different situations (Bandura, 1977). Those beliefs can refer to the appraisal of one's general efficacy or be related to specific tasks and settings. Domain-specific self-efficacies explain how one can perceive their skills to be adequate in one domain but feel less confident about them in another (Zimmerman, 2000). In the academic domain, students' beliefs about their competence to perform tasks are described as academic self-efficacy (ASE; Schunk, 1991). ASE has been identified as a predictor of academic outcomes such as GPA and college retention (Akomolafe et al., 2013; Zajacova et al., 2005). It has also been suggested to be related to academic aspirations and college-going intentions (Gibbons and Borders, 2010).

NFC and ASE have been utilized in tandem to predict the academic performance of college students in a study by Elias and Loomis (2002) who suggested to view ASE as a mediator of the positive relationship between NFC and GPA. This implies NFC to have a positive impact on academic performance *and* on ASE, which in turn has a positive effect on performance. Consequently, assuming a mediated relationship seems promising for the inspection of NFC and ASE's relations to study intention.

### The present study

The present study aims to examine and explain differences in study intention, which we operationalize as the personal intention of high school students to pursue higher academic education, by considering the social variable of educational background in the family as well as the trait variables NFC and ASE. We assume the following:

Students from academic households likely report higher study intention than their peers from non-academic households (H1).

NFC and ASE positively affect study intention (H2 and H3).

ASE mediates the effect of NFC: engagement in and enjoyment of cognitively challenging tasks (NFC) should result in more confidence in one's academic skills (i.e., ASE), which in turn is assumed to positively affect study intention (H4). We reason that even if a student has a high NFC, there are intermediate factors on the pathway that lead them to pursue an academic degree. We suggest that ASE is a mediator that facilitates students with varying levels of NFC to form study intentions, while also being affected by NFC.

**Figure 1:**
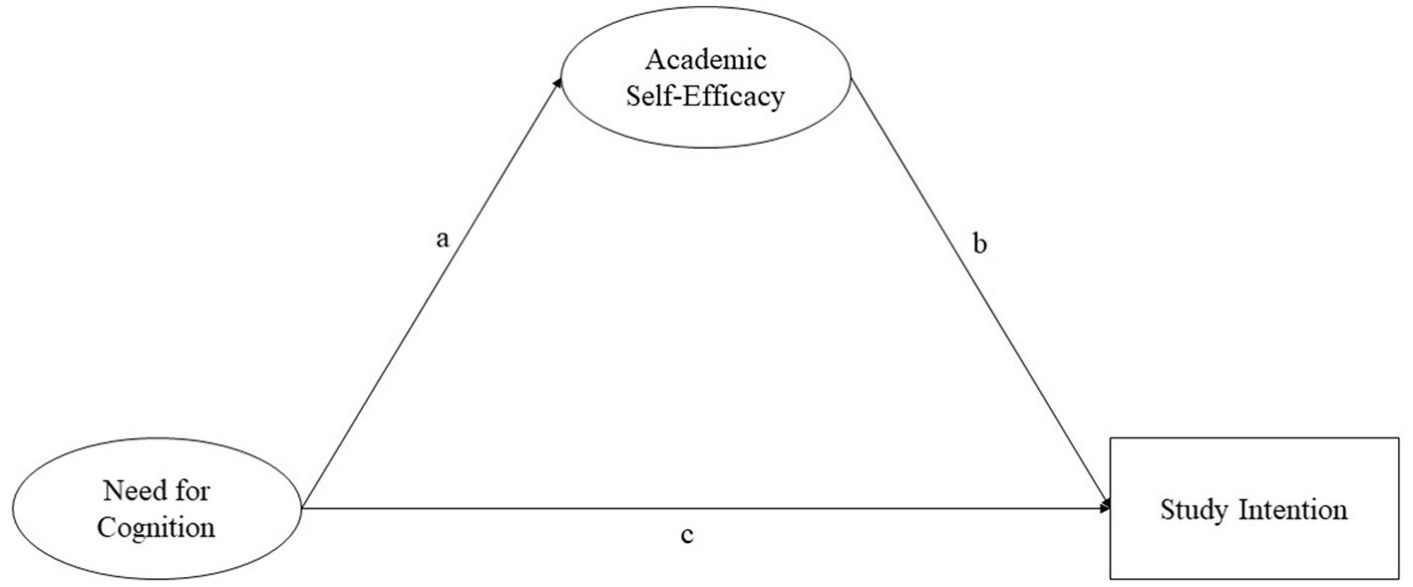
Graphical representation of assumed relationships.

Figure 1 visualizes the assumed relations between students' NFC and ASE and their self-reported study intention. The model proposes a direct effect of NFC on study intention, as well as an indirect effect of NFC on study intention through ASE. A control for student gender is included in the model because women are more likely to enter universities in Germany (Uunk and Pratter, 2021). There has been evidence in past research for associations between academic performance and the traits NFC and ASE (Elias and Loomis, 2002), which is why the potential confounder GPA (school grades) was also included in the model.

The relations between NFC, ASE, and study intention should be invariant for students from different educational backgrounds (H5): While first-generation students have been reported to score lower on ASE, educational aspirations, and, in some instances, NFC, there is no evidence to suggest differences in the interrelations of these variables to each other across groups. This implies that eventual differences in study intention are explainable through the social variable of educational background alone, but that the “psychological mechanisms” of NFC and ASE do not change because of educational background.

## Method

### Procedure

Data were obtained from German high school students in grades 11–13 (the last 3 years of secondary education in Germany) via online survey in the period between July 2020 and January 2021. Participation in the study was completely voluntary.^1^

### Sample

Students in grades 11–13 were invited via mail forwarded by their schools to participate in the online survey. Out of 2,718 participants who followed the link to the online survey, *N* = 1,776 from 46 schools completed the questionnaire. Participation was voluntary and there was no reward for participation. For the purpose of this study, *n* = 149 respondents who did not report their parents' educational background were excluded from the analysis. Further excluded were *n* = 92 participants who were not in grades 11–13 or who had graduated prior to completing the questionnaire. Participants whose replies suggested improbable demographic data (e.g., age older than 22) were also excluded (*n* = 146). Overall, this led to the retainment of *N* = 1,389 students with a mean age of 17.25 years (SD = 1.05 years, range from 16 to 22 years). 69.5% (*n* = 965) of the participants were female.

### Questionnaire

#### Need for cognition

NFC was assessed with the German Short NFC-Scale by Bless et al. (1994), an adaptation of the *Need for Cognition Scale* originally presented by Cacioppo and Petty (1982). Respondents indicated their agreement or disagreement with each statement on a 7-point rating scale ranging from 1 (*strong disagreement*) to 7 (*strong agreement*). Lins de Holanda Coelho et al. (2020) presented a six items short form for the efficient assessment of NFC. In the present study, these six NFC items were retained for all further analyses. Consistent with previous findings on the factor structure of NFC (Chiesi et al., 2018; Hevey et al., 2012), a one-factor model fit the data reasonably well [χ^2^ = 36.234, *df* = 9, *p* < 0.001, CFI = 0.983, RMSEA = 0.047; using the ML estimator in lavaan (version 0.6.18, Rosseel, 2012)]. Score reliability was estimated as ω = 0.84 and α = 0.76 for the present data, using the psych package in R (Revelle, 2024).^2^

#### Academic self-efficacy

ASE was assessed with Mohrenweiser and Pfeiffer's (2016) adaptation of the *Occupational Self-Efficacy Scale* by Rigotti et al. (2008). Agreement to each of a total of six items was indicated on a 4-point scale ranging from 1 (*does not apply*) to 4 (*fully applies)*. Again, a 1-factor model fit the data reasonably well given the inclusion of one correlated pair of residuals in the model specification (χ^2^ = 55.060, *df* = 8, *p* < 0.001, CFI = 0.992, RMSEA = 0.065; using lavaan's wlsmv estimator with Theta parameterization). The shared variance of the two items was likely due to their similar content (“With my past education, I feel well prepared for higher education.” and “I feel prepared for the demands of higher education.”). For ASE, score reliability was estimated as ω = 0.85 and α = 0.82.

#### Study intention

Students indicated their study intention by responding to the statements “I would like to go to college” and “I will go to college.” Agreement was indicated on a 4-point scale, ranging from definite disagreement to definite agreement (*no, rather no, rather yes, yes*). The items were formulated upon the assumption that while some students in grade 11–13 may already have formed definite intentions for their future careers, many students at that age may not yet have done so but can probably indicate a tendency. The two items correlated at *r* = 0.724 (*p* < 0.001). In all models, study intention was operationalized as the average of the two items and thus as a manifest variable.

#### Demographic data

Students were asked to report their age, gender, if and which of their parents or legal guardians had graduated from university (or a comparable higher academic education institution) as well as their current school grades in the subjects Mathematics, English, and German on a scale from 1 to 6, with a 1 representing the highest possible (“best”) grade within the German school system. These subjects were chosen for two reasons: one, they are mandatory for all students (which means grades are available for everyone) and two, the GPA resulting from their average is independent of elective courses which may introduce bias.^3^ GPA was estimated as a latent factor and its variance was fixed to 1, with the three factor loadings for the subjects Mathematics, English, and German constrained to be equal.

#### Other measures

For the purpose of another study, the survey also included a variety of further measures, none of which were relevant or considered for the present study.

### Data analysis

All hypotheses were tested in R. Analysis of variance (ANOVA) was conducted to compare the intention to pursue higher academic education of students from different backgrounds (H1). Structural equation modeling (SEM) analyses were performed (using the wlsmv estimator) to examine the proposed relations between the study variables (H2–H4). We then evaluated potential differences in the relations for students from different educational family backgrounds (mother, father, or any legal guardian) in a multigroup analysis approach (H5). The size of the group of students reporting no parent having obtained an academic degree was *n* = 727. *n* = 337 students indicated one parent possessing an academic degree, and *n* = 325 students reported both parents to possess an academic degree. Table 1 includes a summary of the study variables' descriptive statistics and intercorrelations for the entire sample, and descriptive statistics across groups of parental education can be found in Supplementary Appendix A3.

**Table 1:** Descriptive statistics and correlations for main study variables.

**Variable**	**Min**	**Max**	***M***	**SD**	**1**.	**2**.	**3**.	**4**.	**5**.
1. Study intention	1	4	3.352	0.759					
2. NFC	−2.390	1.701	0.000	0.712	0.348^**^				
3. ASE	−4.068	3.109	−0.008	1.250	0.462^**^	0.479^**^			
4. GPA	1.000	5.000	2.309	0.748	−0.319^**^	−0.354^**^	−0.260^**^		
5. Age	16	22	17.248	1.048	−0.115^**^	0.023	0.064^*^	0.153^**^	
6. Gender	0 (= female)	1 (= male)	965 f (69.5 %)		−0.047^*^	0.102^**^	0.076^*^	0.107^**^	0.076^**^

^**^p < 0.01. ^*^p < 0.05.

NFC and ASE based on CFA factor scores; GPA measured as a latent (smaller values = better GPA), Study intention (larger values = higher study intention) as a manifest variable; all tests two-tailed for non-directed significance testing except for cor (1 [study intention): 6 [gender]), where female > male was assumed for study intention.

## Results

### H1: group differences in study intention

The parental background of students led to significantly different levels of study intention: *F*_(2, 1386)_ = 20.31, *p* < 0.001, $\eta_{p}^{2}=$ 0.028. For the group of students of which both parents possessed higher academic education, the average level of study intention was *M* = 3.552 (SD = 0.642). For the group with one academic parent, *M* = 3.399 (SD = 0.699). Finally, for the group of would-be first-generation students, *M* = 3.241 (SD = 0.813). *Post hoc* tests (Tukey) revealed all three comparisons to be significant (Both vs. One: Δ = 0.158, *p* < 0.01; Both vs. None: Δ = 0.312, *p* < 0.01; One vs. None: Δ = 0.158, *p* < 0.05). This suggests an order in the average study intention of students dependent on their parents' academic education, thus yielding evidence in favor of Hypothesis 1.

### H2 and H3: prediction of study intention

SEM was conducted for the proposed structural model. There were no missing values in the present data set. The mediation model fit the data reasonably well: χ^2^ = 615.073, *df* = 115, *p* < 0.001, CFI = 0.937, RMSEA = 0.056. NFC, ASE, GPA, and gender accounted for a variance of *R*^2^ = 0.314 in study intention, while NFC accounted for a variance of *R*^2^ = 0.367 in ASE. Students who scored high in NFC had higher academic self-efficacy (β = 0.605, *p* < 0.001) and their study intentions were marginally higher (β = 0.060, *p* < 0.06^4^). GPA was correlated with NFC (latent *r* = −0.497, *p* < 0.001) and with academic self-efficacy (latent *r* = −0.090, *p* < 0.01). Students with high GPAs were also more likely to indicate higher study intention (β = −0.198, *p* < 0.001). ASE was a positive predictor of study intention (β = 0.409, *p* < 0.001). Girls indicated a slightly higher study intention as compared to boys (β = −0.047, *p* < 0.05). Together, these results tend to support Hypotheses 2 and 3.

### H4: mediation effect of NFC on study intention through ASE

The total effect of NFC on study intention was significant (standardized coefficient = 0.308, *p* < 0.01), however, the indirect effect through ASE was considerably stronger [standardized indirect coefficient = 0.247 (0.605 ^*^ 0.409), *p* < 0.01] than the direct effect from NFC on study intention (standardized direct coefficient = 0.060, *p* < 0.06). These results suggest ASE to be a mediator of NFC's effect on study intention, which is in support of Hypothesis 4 and previous findings (Elias and Loomis, 2002).

### H5: multigroup model

The multigroup model for the three groups (*no academic parent, one academic parent, two academic parents*) again indicated reasonable fit to the data (χ^2^ = 824.297, *df* = 414, *p* < 0.001, CFI = 0.949, RMSEA = 0.046). We tested for possible parameter equalities in a stepwise fashion and found a model with equal effects across all groups for the mediation paths *a* and *c*, equal *b* paths across groups 2 and 3, and equal effects for GPA and sex across all groups to be tenable. For this model, Table 2 shows the *standardized* coefficients for the direct, indirect, and total effects from NFC to study intention for the three groups. The direct effect of NFC was not significant across groups (*unstandardized* coefficient = 0.049, SE = 0.032, *p* = 0.133). Moreover, the indirect effect of NFC was significant across all groups but stronger for the group with no academic parent as compared with both other groups [unstandardized coefficients of 0.260 (SE = 0.030) vs. 0.170 (SE = 0.023)], a finding which is driven by the effect of ASE on study intention (for group “0”: unstandardized coefficient = 0.265, SE = 0.028, *p* < 0.001; for groups “1” and “2”: 0.174, SE = 0.022, *p* < 0.001). Finally, the amount of variance explained in intention to study was *R*^2^ = 0.317 for group “None,” *R*^2^ = 0.271 for group “One,” and *R*^2^ = 0.299 for group “Both.” Overall, these findings are not supportive of Hypothesis 5. Instead, they suggest that there may be differences in the relations between NFC, ASE, and study intention conditional on family background, notably, for students with no parents (reportedly) possessing any academic degrees.

**Table 2:** Standardized direct, indirect, and total effects from NFC and ASE to study intention across groups of parental education.

		**No parents with academic degree**			**One parent with academic degree**			**Both parents with academic degree**		
		**Effect**	**SE**	***p***	**Effect**	**SE**	***p***	**Effect**	**SE**	***p***
NFC → SI	c	0.050	0.033	0.133	0.057	0.038	0.134	0.062	0.041	0.130
ASE → SI	b	0.443	0.036	< 0.001	0.343	0.040	< 0.001	0.368	0.043	< 0.001
NFC → ASE	a	0.601	0.028	< 0.001	0.584	0.034	< 0.001	0.591	0.034	< 0.001
Indirect	a^*^b	0.266	0.025	< 0.001	0.206	0.027	< 0.001	0.206	0.027	< 0.001
Total	c + (a^*^b)	0.316	0.032	< 0.001	0.256	0.032	< 0.001	0.256	0.032	< 0.01

SI, study intention.

## Discussion

The purpose of this study was to investigate differences in study intentions between students from academic and non-academic households and how NFC and ASE are related to these intentions.

Students from academic households indicated significantly higher study intentions than their peers from non-academic households. NFC and ASE, as well as GPA, were confirmed as positive predictors for high-school students' study intentions (in the case of NFC, at least showing this expected trend). ASE assumed a mediating role in the relation between NFC and ASE. Engagement in and enjoyment of cognitively challenging tasks (NFC) positively impacts the confidence students have in their ability to succeed in academic settings (ASE), which in turn positively impacts academic ambition. These results support the first three hypotheses and past research on the positive effects of NFC in education, as well as the mediating role of ASE (Elias and Loomis, 2002). Congruent with Mussel's (2013) framework of intellect, students who are higher in NFC do indicate higher intentions to *seek* out higher education via the route through ASE.

Contradictory to the fifth hypothesis, the relations between ASE, NFC, and study intention differed contingent on parental background. The direct effect of NFC on study intention was not significant and for students with no academic parent, the mediating effect of ASE was stronger than for members of the other groups, which suggests an increased importance of self-efficacy beliefs rather than “pure” NFC for would-be first-generation students. In their case, pursuing higher education appears to be less favorable as compared to their peers from academic households. This would make high levels in ASE especially advantageous in the group with no academic parents Note that, although not part of our hypotheses, multigroup models for ASE showed evidence of a rising magnitude of ASE from no academic parents to one academic parent to two academic parents (see Supplementary Appendix A2). In addition, the style of thinking associated with higher NFC scores might be less exemplified in non-academic households. In contrast, for students with two academic parents, the pursuit of higher education might be the standard expectation, which could even seem like a burden. Arguably, students who have one parent with an academic and one parent with a non-academic background might experience the “best of both worlds” from a self-determination view, and let their personal preferences weigh heavier when deciding on their own educational goals.

### Limitations and outlook

It seems plausible that the relationships between NFC, ASE, and GPA are reciprocal. Enjoyment of cognitive effort may result in good grades, but good grades may also result in more enjoyment of cognitive effort. A recent study by Strobel et al. (2024), utilizing a longitudinal study design, shows such a dynamic interplay. For the present study, we cautiously argue that its cross-sectional design allows for a meaningful inspection of the assumed relationships between NFC, ASE and study intention because the intentions do not constitute actual behavior but rather a reflection of how an individual sees her or his future academic prospects, where the presently experienced NFC and ASE can act as predictors.

Family status or presence of parents at home might be relevant factors for the impact of parental education. Furthermore, there is no consideration of potential other caregivers, such as older siblings, who could act as role models and provide social support. In addition, the influence of student and parent gender on the effect of parental education has not yet been evaluated. Also, male participants are underrepresented in the present study.

Notably, variance in the variable study intention was fairly limited in this sample. As apparent from the relatively high mean in GPA, respondents were doing rather well in school and the majority was leaning toward the pursuit of an academic degree. GPA and parental education were also self-reported. However, the overall high level of study intentions in this student sample, of which more than half were to-be first-generation students, are in line with the increasing enrollment rates as reported by the German Federal Statistical Office (Destastis) (2025).

One factor potentially affecting our results is the introduction of home-schooling and restrictions to social life during the “COVID-19 pandemic,” which were effective during the period of data collection. Especially, the limited possibilities for social exchange could have impacted differences regarding the fostering of academic ambitions between the three groups.

The implications of this study for education are that disadvantages in the pursuit of higher education appear to persist for students from non-academic household. Thus, it remains important to encourage students' academic self-efficacy beliefs and intellectual curiosity. This is a challenge to be confronted not just by teachers at school, but also within families. To close the educational gap between individuals from different social backgrounds, it appears desirable to design and implement interventions and support systems that target self-confidence in academic settings.

## Data Availability

All data used for hypotheses testing and a corresponding R script are available from https://osf.io/tnj3m/?view_only=514350ea10f443fd9fa80dfcfb719850.

## Footnotes

^1^For more information about the data collection procedure, see Supplementary Appendix A1.

^2^Supplementary Appendix A2 reports the results of measurement invariance analyses.

^3^At the same time, we acknowledge that a “GPA” consisting of these three subjects only is not equivalent to a full-scale GPA featuring all subjects taught in a given term.

^4^*p*-value from results output halved because of directed hypothesis.
