# Supplementary material for: Need for cognition, academic self-efficacy and parental education predict the intention to go to college—evidence from a multigroup study
Source: Front Psychol. 2025 Feb 5;16:1487038. doi: 10.3389/fpsyg.2025.1487038 (PMC11835861; doi:10.3389/fpsyg.2025.1487038)
Supplement: Supplementary file 1 [file Data_Sheet_1.docx]

Need for cognition, academic self-efficacy and parental education predict the intention to go to college – evidence from a multigroup study

**Appendix**

**A-1**

**Further Information on Data Collection Procedure**

Data was obtained from German high school students via online survey. The survey was sent to all schools offering higher education entrance qualification in four federal states of Germany (with permission to conduct the survey from the school administration authorities where necessary). For context, the country’s educational system is based on three school leaving qualifications. Students can earn their general education school leaving qualification after successful attendance of at least nine to ten years of schooling. To qualify for higher education, students are required to complete two to three more years of schooling and completion of the Abitur exams. The degree is not offered by all schools, a system that leads to students attending different school types based on their assumed capabilities after the first four years of schooling. Of relevance for this study were only those schools enabling students to enter higher education. The schools were contacted via E-Mail and their representatives were requested to forward the questionnaire to their students in grades 11 to 13. We accordingly aimed at recruiting all students registered in the contacted schools. However, we have no information concerning the total number of registered students (neither per school nor for the entire group of schools), and therefore, we are unable to calculate a participation quote.

Prior to participation in the study, students and their parents or other legal guardians were informed about general goal of the study. It was announced that a scientific study was to be conducted in order to find out about students’ attitudes towards challenges and how their scholastic education has had an influence on their intellectual development. Participation in the study was voluntary for all schools and their individual students.

Posthoc analysis for achieved power: With the sample size of *N* = 1,389 utilized for our analyses, a posthoc power analysis using semPower (Moshagen & Bader, 2024) yielded an estimated power greater than .99 (for the mediation model specified for testing Hypotheses 2 through 4 and achieving an RMSEA effect size of .56, an alpha level of .05, a sample size of *N* = 1,389, and *df* = 115).

*Reference*

Moshagen, M., & Bader, M. (2024). semPower: General power analysis for Structural Equation Models. Behavior Research Methods, 56, 2901-2922. https://doi.org/10.3758/s13428-023-02254-7

**A-2**

**Items used for the Measurement of Need for Cognition (NFC) and Academic Self-Efficacy (ASE)**

**NFC**

1. Die Aufgabe, neue Lösungen für Probleme zu finden, macht mir wirklich Spaß.

I really enjoy a task that involves coming up with new solutions to problems.

1. Ich würde lieber eine Aufgabe lösen, die Intelligenz erfordert, schwierig und bedeutend ist, als eine Aufgabe, die zwar irgendwie wichtig ist, aber nicht viel Nachdenken erfordert.

I would prefer a task that is intellectual, difficult, and important to one that is somewhat important but does not require much thought.

1. Ich würde lieber etwas tun, das wenig Denken erfordert, als etwas, das mit Sicherheit meine Denkfähigkeiten herausfordert. (-)

I would rather do something that requires little thought than something that is sure to challenge my thinking abilities. (-)

1. Ich trage nicht gerne die Verantwortung für eine Situation, die sehr viel Denken erfordert. (-)

I do not like to have the responsibility of handling a situation that requires a lot of thinking. (-)

1. Denken entspricht nicht dem, was ich unter Spaß verstehe. (-)

Thinking is not my idea of fun. (-)

1. Ich würde komplizierte Probleme einfachen Problemen vorziehen.

I would prefer complex to simple problems.

*Note:* In contrast to Holanda Coelho et al. (2020), we employed three items each with positively (agree) and negatively (disagree) formulated statements.

**Multigroup (Measurement Invariance) Analyses for NFC**

Results from multigroup modeling (for comparing students with no, one, and two academic parents) showed the assumption of strong factorial invariance to be tenable (*χ*^2^ = 62.519, *df* = 47, *p* = .064, CFI = .990, RMSEA = .027). With the group ‘None’ serving as baseline group, the unstandardized latent factor means were estimated as 0.166 (*SE* = 0.061) for group ‘One’ and 0.344 (*SE* = 0.062) for group ‘Both’. Models testing for a) equal (all set to 0) latent factor means across all three groups (*χ*^2^ = 95.130, *df* = 49, *p* < .01, CFI = .971, RMSEA = .045), b) equal (0) latent factor means for groups ‘None’ and ‘One’ (*χ*^2^ = 70.021, *df* = 48, *p* < .05, CFI = .986, RMSEA = .031), and c) equal (0) latent factor means for groups ‘One’ and ‘Both’ (*χ*^2^ = 68.830, *df* = 48, *p* < .05, CFI = .987, RMSEA = .031) suggested that the latent factor means did differ between groups. However, models b) and c) both lent empirical support for the assumption of equal latent factor means for two out of the three groups, but not all.

**ASE**

1. Schwierigkeiten im Studium sehe ich gelassen entgegen, weil ich mich immer auf meine Fähigkeiten verlassen kann.

I am relaxed when I think about difficulties at university because I can always rely on my abilities.

1. Wenn ich im Studium mit einem Problem konfrontiert werde, habe ich bestimmt mehrere Ideen, wie ich damit fertig werde.

When I encounter a problem at university I am sure I will have many ideas how to cope with it.

1. Was auch immer im Studium passiert, ich werde schon klarkommen.

Whatever is going to happen at university, I will be able to handle it.

1. Durch meine vergangenen schulischen Erfahrungen bin ich gut auf ein Studium vorbereitet.

I feel well prepared for university on the basis of my past experiences at (high) school.

1. Ich erreiche die Ziele beim Studium, die ich mir setze.

I will reach my goals at university.

1. Ich fühle mich den Anforderungen eines Studiums gewachsen.

I feel capable of meeting the requirements at university.

*Note*: The original items are in German. Data were collected using these original German items. All items were translated into English by the authors of the present study for the sole purpose of making their content available. These English translations aim at capturing the meaning of the items but only the German items were used.

**Multigroup (Measurement Invariance) Analyses for ASE**

Multigroup analyses yielded acceptable fit for a model assuming strong invariance (we let the correlated pair of residuals vary across groups in this model): *χ*^2^ = 79.228, *df* = 56, *p* < .05, CFI = .996, RMSEA = .030. Again, using group ‘None’ as baseline, the unstandardized latent factor means were estimated as 0.306 (*SE* = 0.091) for group ‘One’ and 0.508 (*SE* = 0.103) for group ‘Both’. Here, models examining a) equal (again, all set to 0) latent factor means across all three groups (*χ*^2^ = 172.033, *df* = 58, *p* < .001, CFI = .982, RMSEA = .065), equal (0) latent factor means for groups ‘None’ and ‘One’ (*χ*^2^ = 96.375, *df* = 57, *p* < .001, CFI = .994, RMSEA = .039), and c) equal (0) latent factor means for groups ‘One’ and ‘Both’ (*χ*^2^ = 96.915, *df* = 57, *p* < .001, CFI = .994, RMSEA = .039) all showed inferior fit compared to the model estimating latent factor means for all three groups, indicating real differences in ASE between all groups.

**A-3**

**Descriptive Statistics across Groups of Parental Academic Education**

| Variable | Min | Max | *M* | *SD* | | |
| --- | --- | --- | --- | --- | --- | --- |
| *Group 1: No Parents with Academic Degree (n = 727)* | | | | | |  |
| Study intention | 1 | 4 | 3.241 | 0.813 | | |
| NFC | -2.390 | 1.701 | -0.093 | 0.707 | | |
| ASE | -4.068 | 3.109 | -0.158 | 1.237 | | |
| GPA | 1.000 | 5.000 | 2.430 | 0.696 | | |
| Age | 16 | 22 | 17.377 | 1.068 | | |
| Gender | 0 (= female) | 1 (= male) | 525 f (72.2 %) | | | |
| *Group 2: One Parent with Academic Degree (n = 337)* | | | | | |  |
| Study intention | 1 | 4 | 3.399 | 0.699 | | |
| NFC | -1.942 | 1.701 | 0.035 | 0.706 | | |
| ASE | -4.068 | 3.109 | 0.045 | 1.246 | | |
| GPA | 1.000 | 5.000 | 2.258 | 0.794 | | |
| Age | 16 | 22 | 17.199 | 1.040 | | |
| Gender | 0 (= female) | 1 (= male) | 227 f (67.4 %) | | | |
| *Group 3: Two Parents with Academic Degrees (n = 325)* | | | | | |  |
| Study intention | 1 | 4 | 3.552 | | 0.642 | |
| NFC | -1.750 | 1.701 | 0.171 | | 0.696 | |
| ASE | -3.614 | 3.109 | 0.273 | | 1.232 | |
| GPA | 1.000 | 5.000 | 2.090 | | 0.757 | |
| Age | 16 | 22 | 17.009 | | 0.964 | |
| Gender | 0 (= female) | 1 (= male) | 213 f (65.5 %) | | | |

*Notes:* NFC and ASE based on CFA factor scores; GPA measured as a latent (smaller values = better GPA), Study intention (larger values = higher study intention) as a manifest variable.
